# Long-Term Follow-Up of Phase I Trial of Oncolytic Adenovirus-Mediated Cytotoxic and Interleukin-12 Gene Therapy for Treatment of Metastatic Pancreatic Cancer

**DOI:** 10.3390/biomedicines12051065

**Published:** 2024-05-11

**Authors:** Aseem Rai Bhatnagar, Farzan Siddiqui, Gazala Khan, Robert Pompa, David Kwon, Shyam Nyati

**Affiliations:** 1 Department of Radiation Oncology, Henry Ford Hospital, Detroit, MI 48202, USA; abhatna1@hfhs.org (A.R.B.); fsiddiq2@hfhs.org (F.S.); 2 Department of Medical Oncology, Henry Ford Hospital, Detroit, MI 48202, USA; 3 Department of Gastroenterology, Henry Ford Hospital, Detroit, MI 48202, USA; 4 Department of Surgery, Henry Ford Hospital, Detroit, MI 48202, USA

**Keywords:** metastatic pancreatic cancer, oncolytic adenovirus, overall survival

## Abstract

The long-term follow-up findings of the phase I trial evaluating the efficacy of oncolytic adenovirus-mediated cytotoxic and interleukin-12 gene therapy in metastatic pancreatic cancer (mPC) seem very promising. The study employed a replication-competent Adenovector in combination with chemotherapy in a dose-escalation format. The trial demonstrated a clinically meaningful median overall survival (OS) benefit, with patients in the highest dose cohort exhibiting an impressive median OS of 18.4 months. This contrasts starkly with patients receiving lower doses who experienced a median OS of 4.8 and 3.5 months, respectively. Remarkably, subject number 10, who received the highest dose, demonstrated an extraordinary survival of 59.1 months, presenting a compelling case for further exploration. Additionally, this patient displayed complete responses in lung and liver metastases, a rare occurrence in mPC treatment. Statistical analyses supported the observed survival benefit. The unprecedented OS results emphasize the potential of this treatment strategy and pave the way for future investigations into this promising gene therapy approach.

## Brief Communication

We are writing with great interest to bring attention to the long-term follow-up findings of our earlier study [1]. The study’s comprehensive exploration of a novel gene therapy approach to treating metastatic pancreatic cancer (mPC) has implications that could potentially significantly impact patient outcomes in the future. The grim statistics surrounding pancreatic cancer make any treatment progress a ray of hope for both patients and the medical community. The median survival of mPC is less than 9 months while 5-year survival is only 3% [2].

Oncolytic virotherapy represents a burgeoning frontier in the realm of cancer treatment. As of now, 36 cellular and gene therapy products have been approved by the United States Food and Drug Administration (US-FDA) out of which about one-third are approved for cancer treatment. These therapies harness either natural or genetically engineered viruses which possess the unique ability to selectively replicate within cancer cells (oncotropism) while sparing healthy tissue. Only oncolytic viruses that retain replication competence can effectively target cancer cells. Within normal cells, the body’s antiviral defenses typically impede viral replication; however, in cancer cells, irregular signaling pathways often disrupt these defenses, allowing oncolytic viruses to proliferate and ultimately destroy malignant cells, liberating viral particles into the surrounding environment. Subsequently, these released viruses can target neighboring cancer cells, exploiting tumor-associated antigens and viral components to provoke potent anti-tumor responses. Oncolytic virotherapy for gene therapy utilizes two distinct categories of viruses. Firstly, there are naturally oncotropic viruses. Oncolytic viruses are nonpathogenic in humans because they either exploit the innate antiviral signaling pathways or host oncogenic signaling pathways. They preferentially move towards tumors because of the cancer cell’s ability in evading immune surveillance, resisting apoptosis, and overcoming translational suppression. These genetically engineered viruses harbor gene mutations that enable them to selectively infect and grow in cancer cells while sparring normal cells [3]. Among these, adenoviruses are widely used in gene therapy due to their ability to lyse tumor cells and stimulate immune responses [4].

Two suicide genes, *Escherichia coli* cytosine deaminase (CD) and herpes simplex virus thymidine kinase (HSV-1 TK), have been extensively studied both in preclinical models and clinical settings. These genes enable the conversion of innocuous prodrugs such as 5-fluorocytosine (5-FC) and ganciclovir (GCV) into cytotoxic agents that interfere with DNA synthesis in rapidly dividing cancer cells, leading to their destruction [5,6,7]. The fascination with interleukin-12 (IL-12)-mediated tumor immunotherapy stems from its extensively documented capacity to stimulate both innate and adaptive immune responses. The expression of IL-12 by specialized antigen-presenting cells triggers a cascade effect on natural killer (NK), CD4+, and CD8+ cells, prompting them to secrete interferon-gamma (IFN-γ) and ultimately CXCL10 [8,9]. Both of these molecules harbor multifaceted anti-tumor properties and foster a concerted anti-tumor immune response. A significant advantage of employing oncolytic adenoviruses lies in their capacity to transform “immunologically cold” tumors into “immunologically hot” ones. Pancreatic tumors, often characterized as “cold” due to their lack of infiltrating immune cells and low mutational burden, stand to benefit from this therapeutic paradigm shift [10].

After years of dedicated research in gene therapy at our institution, significant strides have been made in the evolution of adenoviral vectors for suicide gene therapy despite many challenges faced including tumor heterogeneity, targeting specificity (especially for metastatic cells), unpredictable immune response, delivery challenges, transient or short-lived effects, resistance, and safety concerns regarding insertional mutagenesis. This journey has seen advancement through three generations. The pioneering replication-competent adenoviral vector, Ad5-CD/TKrep, represented a significant breakthrough. This vector possessed the ability to not only replicate in tumor cells but also carried two different suicide genes, bacterial cytosine deaminase (CD) and HSV1 thymidine kinase (HSV-TK). This vector exhibited superior efficacy and safety and allowed for combining radiotherapy for increased efficacy due to the conversion of inactive prodrugs into active agents by CD and TK. The upgraded second-generation adenovirus featured enhancements in its enzyme components, including cytosine deaminase from yeast (yCD), a mutant HSV-TK with superior catalytic activity, and the adenovirus death protein (ADP), which allowed for higher oncolytic capabilities. This advancement laid the groundwork for the development of the third-generation vector, Ad5-yCD/mutTKSR39rep-mIL12, which carried dual suicide genes alongside mouse IL-12 [10]. This vector has shown promising results in preclinical models, demonstrating improved tumor control and survival rates compared to earlier versions lacking IL-12 [11]. Additionally, delivering two suicide genes with IL-12 by a single adenoviral vector demonstrated potential in preclinical studies, which supported its further development for human trials [12].

Building upon this foundation, we initiated a phase I clinical trial using replication-competent Ad5-yCD/mutTKSR39rep-hIL12 adenovirus (Ad5-vector) expressing yCD/mutTKSR39 (yeast cytosine deaminase/mutant S39R Herpes Simplex Virus-1 thymidine kinase) and human IL-12 in combination with oral cytotoxic prodrug therapy. This was a single-site, nonrandomized, dose-escalation phase I trial of a third-generation replication-competent adenovirus bearing two suicide genes, HSV-TK and yCD, and an IL-12 expression cassette for the treatment of mPC patients. This phase I study marks a pioneering endeavor on two fronts: firstly, the integration of IL-12 immuno-gene therapy alongside oncolytic adenovirus suicide gene therapy; and secondly, the application of this therapeutic combination in the context of metastatic disease for the first time. By employing oncolytic adenoviruses to induce cancer cell death and IL-12 to incite immune cell infiltration into the tumor microenvironment, this approach aims to reverse the immunologically barren landscape typical of pancreatic cancers. The trial was designed for a stepwise increase in dosage and determination of the highest safe level (phase I). The protocol was sanctioned by the Institutional Review Board, and the research adhered to the principles outlined in the Declaration of Helsinki and the International Conference on Harmonization Good Clinical Practice guidelines. Each participant provided written consent and retained the right to withdraw from the study at any point. Inclusion criteria were patients who were 18 years or older with treatment-naive histologically proven metastatic pancreatic cancer. Patients who were non-metastatic and had certain medical conditions such as concurrent second malignancies were deemed ineligible.

Twelve patients with mPC were enrolled between October 2017 and May 2019. Metastatic lesions were identified in the liver across all subjects. Additionally, there were metastases seen in other organs such as the kidney, lung, spleen, and adrenal gland. Baseline radiological imaging, including CT or MRI scans and lab work was conducted prior to treatment initiation. Treatment commenced with each subject receiving a single endoscopic ultrasound-guided intratumoral injection of the Ad5-vector into the primary pancreatic tumor. Patients received increasing doses of the Ad5-vector [cohort 1: 1 × 10^11^ virus particles (vp), *n* = 3; cohort 2: 3 × 10^11^ vp, *n* = 3, and cohort 3: 1 × 10^12^ vp, *n* = 6] and received 5-fluorocytosine (5-FC, 150 mg/kg/day orally for 7 days) two days after adenoviral injection. Two weeks after the completion of the 5-FC prodrug therapy course (around 21 days after adenoviral injection), subjects initiated chemotherapy at the discretion of the treating physician. The primary endpoints were maximum-tolerated dose (MTD) and dose-limiting toxicities up to day 21. The study design permitted the endpoint to be reached (day 21) before the initiation of chemotherapy to facilitate toxicity assessment and management. Toxicity assessments were conducted weekly before the initiation of chemotherapy, spanning the first three weeks of the study. Subsequently, scheduled follow-up visits with imaging and lab work were mandated at 3, 6, 9, 12, 18, and 24 months post-treatment. Toxicities were graded according to the Common Terminology Criteria for Adverse Events (CTCAE) version 4.03. The secondary endpoint was the rate of grade 3 adverse events, and exploratory endpoints were the viral distribution and level of immunological cytokines to evaluate immune system activation. Blood samples were collected on the day of adenovirus injection before the procedure (day 1) and subsequently on specified days (2, 4, 7, 14, and 21). We evaluated immune system activation by analyzing the serum levels of IL12, IFNγ, and CXCL10 by ELISA. Additionally, peripheral blood mononuclear cells were isolated and were stained with antibodies targeting CD3, CD56, CD4, CD8, CD69, CD45 (RO), Ki67, and Tim3, and then examined using flow cytometry.

All but one patient received at least three cycles of chemotherapy. Patients with favorable performance scores were offered FOLFIRINOX treatment for as many cycles as tolerated. Six patients began with FOLFIRINOX, while five received alternative chemotherapy options (like Gemcitabine/Albumin-bound Paclitaxel). Unfortunately, one subject (patient 3) passed away after 1.6 months due to disease progression following the administration of the adenovirus but before chemotherapy could commence. Approximately 94% of the 121 adverse events observed were grade 1/2, requiring no medical intervention. The study maximum tolerated dose (MTD) was not reached. Hypotheses can be formulated based on these findings that since the MTD was not reached, there remains a possibility that Ad5-vector can be safely administered at higher doses, such as 3 × 10^12^ vp. Primary and secondary outcomes have been previously reported in detail [1]. The increases from baseline in blood serum levels of CD45, CD69, Ki67, and Tim3 have been previously reported, and so have the peak serum immunological cytokine concentrations (IL-12, interferon-gamma (IFNγ), and CXCL10).

While this was not the primary endpoint of the study, an overall survival (OS) analysis was conducted on 8 February 2024. The updated follow-up data showed that higher doses (1 × 10^12^ vp, cohort 3) of the Ad5-vector gene therapy virus provided a clinically meaningful median OS benefit of 18.4 months (range, 3.5–59.1 months) compared to 4.8 months (range, 1.6–5.4 months) and 3.5 months (range, 2.7–10.5 months) for patients receiving low doses of adenovirus, 1 × 10^11^ vp (cohort 1) and 3 × 10^11^ vp (cohort 2), respectively. All the patients in cohorts 1 and 2 had no response to the intratumoral injection of Ad5-vector [all had stable disease (SD) per RECIST 1.1 criteria at the primary site]. For patients in higher dose cohort 3, three had a partial response (PR) and three had SD at the primary site. Subject number 10 is still surviving at close to 5 years (59.1 months), which is truly remarkable and warrants further exploration. This subject had an excellent response to Ad5-vector with the maximum reduction in the size of the primary tumor (70% decrease) of all patients. Moreover, the subject initially had lung and liver metastasis which completely disappeared. None of the other patients had a complete response (CR) at metastatic sites. CA 19-9 levels showed a variable response to treatment. Two patients had normal pre-treatment CA 19-9. Most of the other patients showed an eventual increase in CA 19-9 levels before death. For subject number 10, CA 19-9 levels soon reached normal levels after initiating gene therapy. Molecular analysis was planned but could not be performed because of insufficient tissue samples.

**Figure 1:**
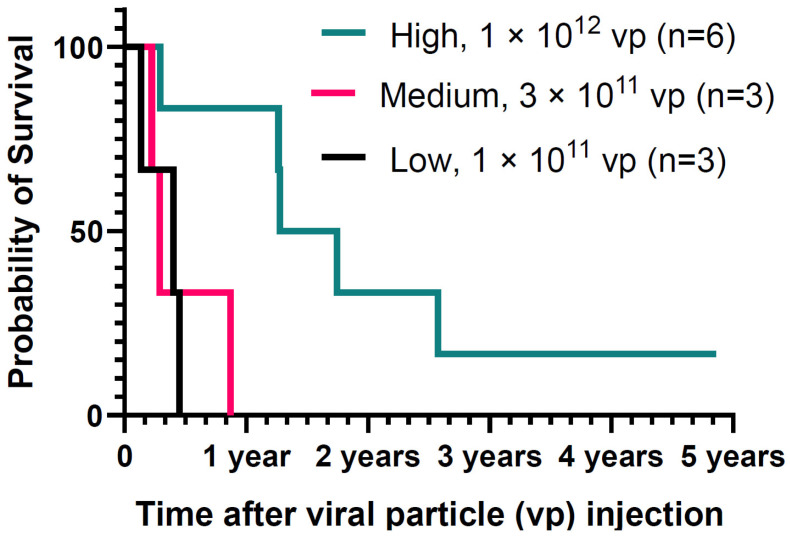
Survival plot.

The log-rank (Mantel–Cox) test *p*-value was 0.01, while the log-rank test for the survival trend was 0.009 (see survival plot, Figure 1). Overall survival was calculated from Ad5-vector injection to the date of death, and patients who were alive at the end of the study period were censored. Kaplan–Meier estimates and the corresponding 95% confidence intervals were reported for OS, as well as 1-, 2-, 3- and 5-year survival probabilities were reported (Table 1). A *p*-value < 0.05 was used for statistical significance. SPSS version 28 (IBM Corp. Released 2021. IBM SPSS Statistics for Windows, Version 28.0. IBM Corp., Armonk, NY, USA) was used for all statistical analysis. Median follow-up for this study population was 0.66 (0.14–4.90) years, while the median OS for the study population was 0.45 (0.00–1.25) years. The median OS by dose group was 0.40 years (range 0.00–0.82) for the low dose cohort, 0.29 years (range 0.19–0.39) for the medium dose, and 1.28 years (range 0.70–1.85) for the high dose cohort with log-rank *p*-value = 0.019. The survival probability of study subjects was compared with the metastatic Pancreatic Cancer SEER database survival rate for the age group 65–70 years, since the median age of study subjects in this trial was 68 years. The 1-year survival probability in the SEER data is 18.9%, while it was 41.7% in our study population (overall) and 83.3% in subjects in cohort 3 (high dose cohort). The 4-year survival probability in the high dose cohort was 15.2%, while in the SEER dataset, it was 2.9% (Table 1) for this age group. Clinical outcomes for patients are listed in Table 2. To our knowledge, this is the first phase I trial that showed a median OS of around 18 months for mPC patients. The observed increase in OS coupled with good primary site radiographic response in three patients in the high dose cohort 3 underscores the potential of this promising treatment strategy. Furthermore, the study’s approach to measuring immune system activation and tumor response through various parameters adds depth to our understanding of the treatment’s mechanisms and potential avenues for improvement. Noteworthy observations from this study include the transient elevation of all the studied serum cytokine levels, including IL-12, IFNγ, and CXCL10, following adenovirus administration. Additionally, this study identifies new ground by treating patients with metastatic pancreatic cancer, a departure from previous trials that primarily focused on newly diagnosed or locally recurrent disease. The possibility of inducing systemic antitumor immunity holds promise for impacting disseminated tumors, particularly those located in the liver and lungs, as evidenced by the outcomes observed in cohort 3 of this study.

Recently, Musher et al. [13] evaluated the safety of oncolytic adenovirus LOAd703 with chemotherapy for advanced pancreatic cancer in a phase I trial. Like our trial, their combination treatment was generally well-tolerated and the maximum-tolerated dose (MTD) was not reached. Immunological responses were observed in most patients, with increased levels of specific T cells. Objective responses were seen in 44% of evaluable patients. The study concluded that combining LOAd703 with nab-paclitaxel plus gemcitabine is feasible and safe in patients with advanced pancreatic ductal adenocarcinoma. This outlines an approach like ours that combines the delivery of an immunostimulatory gene by an oncolytic adenoviral vector in advanced pancreatic cancers which holds potential for therapeutic benefits. In another recent phase I trial by Garcia-Carbonero et al. [14], the authors investigated the safety, maximum-tolerated dose (MTD), and recommended phase II dose (RP2D) of VCN-01, an oncolytic adenovirus designed to replicate in cancer cells with dysfunctional RB1 pathways. The study focused on patients with advanced cancer, particularly pancreatic adenocarcinoma. In the first part, patients with refractory solid tumors received a single dose of VCN-01, while in parts II and III, patients with pancreatic adenocarcinoma received VCN-01 in combination with nab-paclitaxel plus gemcitabine. Results showed an acceptable safety profile, with the MTD and RP2D determined as 1 × 10^13^ viral particles (vp)/patient in Part I, and 3.3 × 10^12^ vp/patient in Part II. Notably, no dose-limiting toxicities were observed in Part III, with an RP2D of 1 × 10^13^ vp/patient. In patients with pancreatic adenocarcinoma, the overall response rate was 50% in both Parts II and III. Viral genomes of VCN-01 were detected in tumor tissue, indicating successful intravenous delivery and replication. Increased immune biomarkers suggested immune activation after VCN-01 administration. The authors concluded that treatment with VCN-01, particularly in combination with nab-paclitaxel plus gemcitabine, showed feasibility, safety, and promising biological and clinical activity in patients with pancreatic adenocarcinoma. A recent comprehensive review by Taylor et al. identified six clinical trials that used oncolytic adenoviral vectors in pancreatic cancer [15]. They concluded that oncolytic adenoviruses elicited clinical responses with manageable side effects, even at high doses. Intravenous administration showed comparable tolerability to intratumoral injections. These trials reflect the safety and potential efficacy of conditionally replicative adenoviruses in pancreatic cancer treatment, particularly through intravenous delivery, paving the way for further investigation and hopeful advancements against this formidable disease.

In conclusion, the encouraging results from the long-term outcomes of this phase I gene therapy trial provide a beacon of hope for mPC patients. This will lay a solid foundation for the next phase of clinical evaluation. We look forward to witnessing the potentially transformative impact of Ad5-vector in the ongoing fight against mPC in a phase II/III clinical trial soon. In this study, we plan to study the effect of gene therapy on immune system activation and tumor response in more detail. This will be carried out using the patient’s blood (via a liquid biopsy technique) and the tumors harvested at/after the gene therapy injection. Total cytokine/chemokine array, immune cells assay (CyTOF), circulating tumor cells/circulating tumor DNA (ctDNA), targeted next-generation sequencing (NGS), and viral replication will be measured from liquid biopsies while tumor-infiltrating lymphocytes, tumor growth (Ki67 assay), and the replication of viral particles (electron microscopy) will be assessed in tumors.

## Figures and Tables

**Table 1:** Comparison of the survival probability of the study subject in this phase I trial with that of overall SEER survival (age group 65–70 years).

Survival Probability	Overall Study Population	Low Dose Group	Medium Dose Group	High Dose Group	SEER Data (Age 65–70 Years)
1 year	41.7%	0%	0%	83.3%	18.9%
2 year	16.7%	-	-	33.3%	7.3%
3 year	8.3%	-	-	15.2%	4.3%
4 year	8.3%	-	-	15.2%	2.9%
5 year	-	-	-	-	2.4%

**Table 2:** Patient outcomes overview.

Patient Number	Radiographic Size				RECIST 1.1 Response		Chemotherapy	Overall Survival (Months)	Status
	Before Ad5-Vector Injection		After Ad5-Vector Injection		Primary Site	Metastatic Site			
	Primary Site (cm)	Largest Metastatic Site (cm)	Primary Site (cm)	Largest Metastatic Site (cm)					
1	3.6	1.6	4.3	1.8	SD	SD	FOLFIRINOX	4.8	Died
2	4.3	3.4	4.3	3.5	SD	SD	G/A	5.4	Died
3	5.0	5.6	5.0	5.6	SD	SD	None	1.6	Died
4	3.6	3.4	3.6	2.9	SD	SD	G/A	2.7	Died
5	2.8	3.2	2.0	3.7	SD	SD	FOLFIRINOX, G/A	10.5	Died
6	3.9	1.3	4.4	2.1	SD	PD	FOLFIRINOX	3.5	Died
7	2.5	1.7	2.8	1.9	SD	SD	G	15.2	Died
8	2.7	0.5	2.8	3	SD	PD	G/A, FOLFIRI, G/C	30.9	Died
9	5.0	2.0	4.7	8.5	SD	PD	FOLFIRI	3.5	Died
10	3.3	1.8	1.0	Not seen	PR	CR	FOLFIRINOX, X	59.1 *	Alive
11	5.9	3.5	3.4	4	PR	SD	FOLFIRINOX, G/A, G/C	20.9	Died
12	4.4	5.2	2.8	6.6	PR	PD	FOLFIRI, FOLFOX, G/A	15.3	Died

Abbreviations: CR, complete response; G, Gemcitabine; G/A, Gemcitabine/Albumin-bound Paclitaxel; G/C, Gemcitabine/Cisplatin; PR, partial response; RECIST, Response Evaluation Criteria in Solid Tumors; SD, stable disease. * At the time of submission.

## Data Availability

All data are incorporated into the article.
